# LC-MS metabolomics profiling of *Salvia aegyptiaca* L. and *S. lanigera* Poir. with the antimicrobial properties of their extracts

**DOI:** 10.1186/s12870-023-04341-5

**Published:** 2023-06-26

**Authors:** Alyaa Nasr, Israa Yosuf, Zaki Turki, Ann Abozeid

**Affiliations:** Department of Botany and Microbiology, Faculty of Science, Menoufia University, Shebin Elkoom, 32511 Egypt

**Keywords:** Antibacterial, Antifungal, LC-MS, *Salvia aegyptiaca* L, *Salvia lanigera* L

## Abstract

**Background:**

*Salvia* L. (Lamiaceae) found in almost all countries in temperate and tropical regions. Both* S. aegyptiaca* L. and *S. lanigera* Poir. have a rather wide distribution in Egypt (Mediterranean region, Gebel Elba and nearly the whole Sinai). *Salvia *species showed antibacterial and antifungal activities against several groups of food microorganisms and pathogens, so they are considered as a natural foods preservatives.

**Aim:**

Investigate the phytochemical profiles of *S. aegyptiaca* & *S. lanigera* collected from their natural habitats in Egypt and test the antimicrobial activities of both species against some bacteria and fungi pathogenic strains.

**Methodology:**

In the present study, *S. aegyptiaca* and *S. lanigera* were collected from their natural habitat. Total phenolics and flavonoids contents were measured for aerial parts of both *Salvia* spp.. The separation and identification of the pure active materials of both *Salvia* sp. by using LC-MS system (UHPLC-TSQ Quantum Mass Spectrometer). The antimicrobial activities of the ethanol, water and benzene extracts of the two species were tested against different pathogenic strains and compared with the standard antimicrobial drug (Gentamycin). Antimicrobial activity was determined by using agar disk diffusion method.

**Results:**

The phenolics content in *S. lanigera* 132.61±6.23 mg/g and *S. aegyptiaca* 125.19±4.97 mg/g, while the flavonoids content was 35.68±1.84 and 40.63±2.11 mg/g, respectively. Through LC-MS analysis, two compounds were detected in both species; heptadecanoyl coenzyme A, that the highest percentage (13.5%) in *S. aegyptiaca* and (11.5 %) in *S. lanigera*. Oenin, in a peak area of 3.1% in *S. aegyptiaca *and 1.2 % in *S. lanigera*. Ethanol extract of the two species had the most inhibitory effect against all tested microorganisms that exceeded the effect of the standard, except for* Mucor reinelloids* which was more sensitive to the water extract. Moreover, *S. lanigera* ethanol extract showed larger inhibition zone than *S. aegyptiaca* in all tested microorganisms except for *Pseudomonas aeruginosa*.

**Conclusion:**

This study shows the important phytochemicals that improve the antibacterial and antifungal activities of *Salvia aegyptiaca* and *S. lanigera*.

**Supplementary Information:**

The online version contains supplementary material available at 10.1186/s12870-023-04341-5.

## Background

Wild plants are considering a reservoir of phytochemicals that have been investigated against various animal and human pathogens however; few studies are interested with their effect on fungal plant pathogens [1]. Botanical fungicides are attracting global attention as safer alternatives to chemical one with potentially less risk to humans and environment. The genus *Salvia* is the largest in the sage family (Lamiaceae), with nearly 1000 species of shrubs, herbaceous perennials and annuals. The species of *Salvia* L. originated from South America and found in almost all countries of temperate and tropical regions [2].

**Fig. 1:**
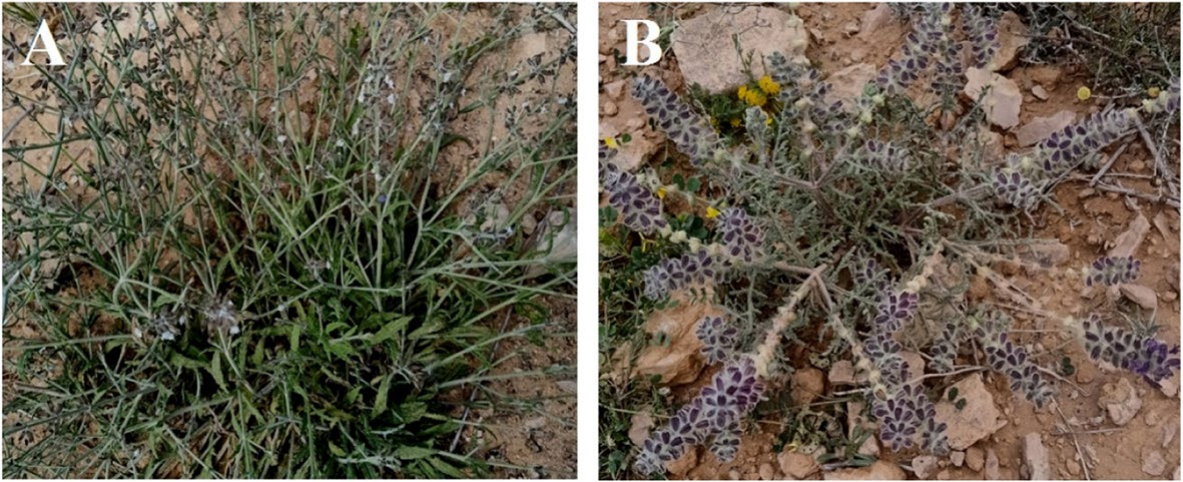
Habitof both studied *Salvia* species **A** *Salvia aegyptiaca*, **B** *Salvia lanigera*

In Egypt, nine *Salvia* species were recorded including *S. aegyptiaca* L. (Egyptian sage) and *S. lanigera* as herbaceous, xerophytic perennials [3] (Fig. 1). However, *Salvia* L. plants are easy-to-grow herbs. Besides, there are a lot of studies mentioned the allelopathic and phytoremediation effects of *Salvia* species [4–6].

There is an increasing global interest in the use of *Salvia* L. species as shown by many studies conducted on the plant in recent years; as many *Salvia* species are used in cosmetics, perfumes and pharmaceutical industries; in addition, some species are grown as ornamental plants [7]. Seeds from some *Salvia* species are used as a traditional food in central and southern America [8], in addition, some *Salvia* species are used as food plants by the larvae of some *Lepidoptera* (butterfly and moth) [9]. Beside the economic importance, *Salvia* species have been used since ancient times in traditional medicines around the world. They possess antioxidant, antidiabetic, antitumor, antiplasmodial and anti-inflammatory activities [10].

*Salvia* species showed antibacterial and antifungal activities against several groups of food spoilage microorganisms and food borne pathogens, so they are considered as useful natural preservatives to improve the safety of foods [11, 12].

Egyptian sage *S. aegyptiaca* is used for various medicinal and cosmetic purposes [13]. The seeds are used as a demulcent and to treat diarrhea and hemorrhoids [14]. The whole plant has been used against gonorrhea and eye diseases, and as an antiseptic, cicatrizant, antispasmodic and stomachic [15]. It has also been mentioned to be significant in cases of nervous disorders, dizziness, trembling and for stopping perspiration [16]. The extracts of *S. aegyptiaca* showed an antioxidant, anti-Alzheimer and antidiabetic enzymes inhibition activities [17]. *S. aegyptiaca* contain the monoterpene derivatives, tricyclene, limonene, β-pinene, caryophyllene oxide and β-caryophyllene [18].

*S. lanigera* has been used for the treatment of human inflammatory diseases [19]. Its extracts were reported to have antioxidant activity, cytotoxic activity on human carcinoma cell line, β-carotene bleaching, anti-neurodegenerative activity, tyrosine inhibitory activity [20] and an antitumor activity [21]. L-ascorbic acid, tert- butyl-4- hydroxy toluene, gallic acid, thymol, hexadecanoic acid, carvacrol and a-thujone have been found in *S. lanigera* [11].

The antimicrobial effects of *S. aegyptiaca* and *S. lanigera* were tested and showed variations among different extracts [11, 20–24]. In Egypt, there is no previous complete work on antimicrobial properties of *Salvia* species, that what encourage us for studying these taxa.

## Results

### Phytochemical Study

#### Total phenolics and flavonoids contents

The total phenolic content of the aboveground parts of *S. lanigera* (132.61 ± 6.23 mg GAE/ g dry extract), was higher than that of *S. aegyptiaca* (125.19 ± 4.97 mg GAE/ g dry extract).

whereas the total flavonoids content of *S. aegyptiaca* (40.63 ± 2.11 mg QE/ g dry extract), was higher than its content in *S. lanigera* (35.68 ± 1.84 mg QE/ g dry extract) (Table 1).

**Table 1:** Total phenolics and flavonoids contents in *Salvia* sp

Sample Code	Total Phenolics (mg GAE/ g dry extract)	Total Flavonoids (mg QE/ g dry extract)
*S. lanigera*	132.61 ± 6.23	35.68 ± 1.84
* S. aegyptiaca*	125.19 ± 4.97	40.63 ± 2.11

Total phenolics and flavonoid contents were expressed in gallic acid equivalent (GAE mg/g) and quercetin equivalent (QE equiv/mg)

#### LC-MS analysis of phytochemicals

**Fig. 2:**
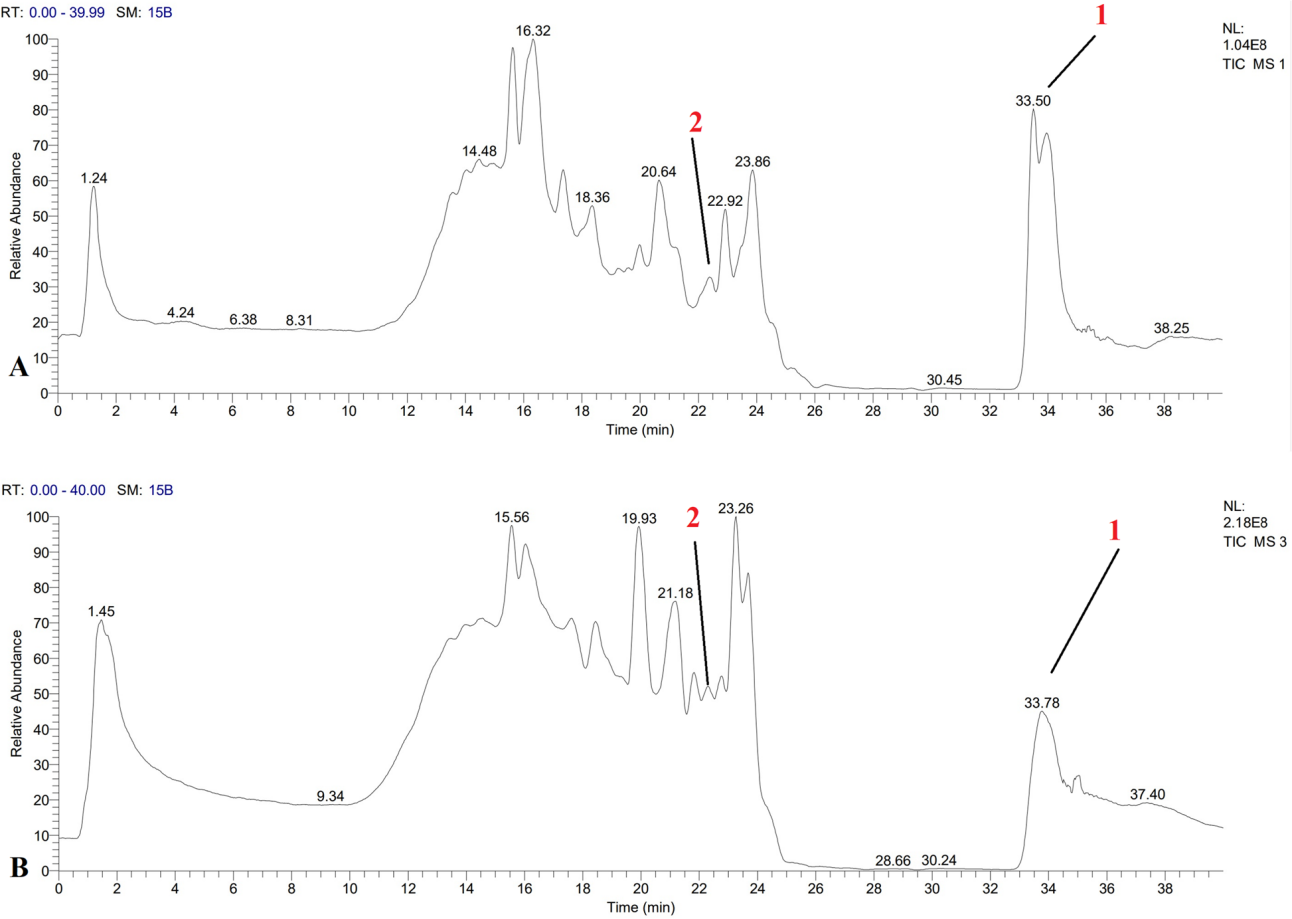
LC-MS analysis of active compounds in extract of: **A** *S. aegyptiaca *L. and **B** *S. lanigera *L., 1 Heptadecanoyl coenzyme A, 2 Oenin

Seventeen phytochemicals were identified through LC-MS analysis in the extract of *S. aegyptiaca*; tow terpenoids, one flavonoid, four phenolics, five alkaloids, tow steroids, one mercaptans and two amine compound; while fifteen phytochemicals were identified in the extract of *S. lanigera;* one terpenoids, one flavonoids, five phenolics, four alkaloids, one mercaptans and three amine compounds (Appendix, Table 2; Fig. 2).

**Table 2:** Compounds identified by LC-MS in *S. aegyptiaca* (*S.a.*)& *S. lanigera* (*S.l.*) extracts; retention time (RT), molecular weight (M.wt.)

	Peak area%						
Compound name	*S.a.*	*S.l.*	RT	M. formula	M.wt.	Plant have this comp.	References
**Terpenoids**							
Phenazine,2,2’-(3,7,12,16-tetramethyl-1,3,5,7,9,11,13,15,17-octadecanonaene-1,18-diyl)bis[3,4-dihydro-1,3,3-trimethyl-, (all-E)-	-	**6**	13.38	C52H56N4	736		
Carnosol	**5.8**	-	20.64	C20H26O4	330	Labiatae herbs, such as *Rosmarinus officinalis* (rosemary) and *Salvia officinalis* (sage)	[25, 26]
Psi.,.psi.-Carotene,3,3’,4,4’-tetradehydro-1,1’,2,2’-tetrahydro-1,1’-dimethoxy-2,2’-dioxo-	4.9	-	20.75	C42H56O4	624	Purple photosyntheticpigmentin *Rubrivivax gelatinosus*, *Glycyrrhiza glabra*	[27, 28]
**Flavonoieds**							
Ononin	**7.4**	-	16.3	C22H22O9	430	Soybean (leaves) *Glycyrrhiza uralensis* (roots)	[29, 30]
Ginkgetin	-	3.5	13.89	C32H22O10	566	*Ginkgo biloba (leaves)* *Selanginella moellendorfii*	[31, 32]
**Phenolics**							
Vitamin K1	**13**	-	1.47	C31H46O2	450	Leaves of *Brassica* sp., Leaves of Spinach,Carrot, Hazelnuts, Grapes, Tomato, Zucchini, Potato	[33, 34]
Aloin	**9.6**	-	29.32	C21H22O9	418	The exudate of 68 Aloe species	[35]
Pentacontanoic acid, propyl ester	-	3	21.81 34.63 34.98 3.42	C53H106O2	774	*Dolichandra cynanchoides* (leaves) *Salix viminalis* (leaves) *Sterculia setigera* (root)	[36, 37]
Malvidin 3-O-galactoside cation or Oenin	3.1	1.2	22.26 22.32	C23H25O12	493	*Padus virginiana* (leaves), Berries (violet, purple or red fruit), Pomegranate (red fruit), Red grape (red fruit)	[38, 39]
2á,4a-Epoxymethylphenanthrene-7-methanol,1,1-dimethyl-2-methoxy-8-(1,3-dithiin-2-ylidene) methyl-1,2,3,4,4a,4b,5,6,7,8,8a,9-dodecahydro-, acetate	-	2.4	17.67	C27H38O4S2	490	*Grape seeds* (0.32%) *Papaver rhoeas* (0.24%)	[25, 40]
Pentacontanoic acid, ethyl ester	1	-	22.38	C52H104O2	760	*Dolichandra cynanchoides*(leaves) *Salix viminalis* (leaves) *Sterculia setigera* (root)	[36, 37]
Leucrose, Perboryliert	-	0.8	37.61	C44H94B8O11	886	*Pogostemon cablin* (Lamiaceae)	[41]
Olein, 3-palmito-2-stearo-1-	-	0.5	24.42	C55H104O6	860		
**Alkaloids**							
1,2-Di(2,3,7,8,12,13,17,18-Octaethyl-7,8-dihydro-21 H,23 H-porphinyl-10)ethane	-	**10.19**	19.9	C74H98N8	1098		
6à,7à:14,7á-Bis(oxymethylene)-4,5à-epoxy-17-methylmorphinan-3-ol	-	**5.4**	15.56	C19H21NO4	327	*Papaver somniferum* (Opium poppy)	[42]
Acepromazine	4.1	-	15.62	C19H22N2OS	326		
5-Isoxazolidinol, 2,3-diphenyl-,acetate (ester), cis	-	3.6	18.45	C17H17NO3	283	*Croton sparsiflorus* (whole plant) *Monodora junodii*	[43, 44]
3-[7,12-Bis-(2-hydroxy-ethyl)-18-(2-methoxycarbonyl-ethyl)-3,8,13,17-tetramethyl-porphyrin-2-l]-propionic acid methyl ester	2.6	-	14.42	C36H42N4O6	626		
Dimethyl-fenbend azole	2.1	-	15.56	C17H17N3O2S	327	*Terminalia chebula, Butea frondosa*, *B. superba, Syzygium aromaticum*, *Chlorophytum borivilianum*, *Mucuna pruriens*	[45, 46]
2-Benzyl-3-(4-methoxyphenyl)-3,4,4a,7-tetrahydroisoquinolin-1(2 H)-one	-	1.6	14.46	C23H23NO2	345	*Murraya koenigii* (Curry tree leaves)‏	[47]
Tetrandrine	1.1	-	19.94	C38H42N2O6	622	*Stephania tetrandra* and other Chinese and Japanese herbs	[48]
Wilforine	0.7	-	0.13 3.09 6.46	C43H49NO18	867	*Tripterygium wilfordii* (dried roots) *Monteverdia laevis*	[49, 50]
**Steroids**							
Cholest-7-en-6-one, 2,3,14,20,22,25-hexahy droxy-, (2á,3á,5á,22R)-	2.6	-	17.35	C27H44O7	480	*Cyanotis vaga* *Ajuga turkestanica Rhaponticum carthamoides*	[51, 52]
5à-Cardanolide, 3á,14,19-trihydroxy-	0.85	-	38.16	C23H36O5	392	flowers and leaves of *Digitalis purpurea, D. orientalis* and *D. lanata* (foxgloves)	[53]
**Mercaptans**							
Heptadecanoyl coenzyme A	**13.5**	**11.5**	33.9	C38H68N7O17P3S	1019		
**Amine compounds**							
B 1 (BINDER)	-	**10.1**	23.66	C72H124N4O17	1316	Cassava plant (roots), Potatoes and another starches plants	[54]
Rifabutin	-	**10.5**	23.26	C46H62N4O11	846		
Ferrioxamine	**9.6**	-	23.85	C27H45FeN6O9	653	all of plants that contain Fe ions	[55]
3’,5’-Cyclic Inosinemonophosphate	-	**7**	16.01	C10H11N4O7P	330		
Vitamin B12	**7.1**	-	23.76	C63H88CoN14O14P	1354	Soybeans, Seaweed (*Pyropia* sp., *Ulva* sp.and *Monostroma* sp., *Porphyra yezoensis*, *Chlorella vulgaris*), Nutritional yeast	[34]

Two compounds were detected in both species; heptadecanoyl coenzyme A; a dervitative of the fatty acid heptadecanoic acid that represent the largest peak areas 13.5% and 11.5% in *S. aegyptiaca* and *S. lanigera*, respectively. It has a role as a cellular energy metabolite [38]. Play vital roles in plants as a component of lipid membranes that demarcate cells and organelles, as sources of stored energy in the form of neutral lipids, and as signaling molecules that elicit plant responses to adverse conditions [39]. Oenin; a pigment that was detected before in *Padus virginiana* leaves, red fruit of Berries, Pomegranate and Red grape [56, 57]; represent a peak area of 3.1% and 1.2% in *S. aegyptiaca* and *S. lanigera*, respectively.

The highest compounds in *S. aegyptiaca* and *S. lanigera* were Carnosol (5.8%), it is a diterpenoid that has anticancer, anti-inflammatory, chemo- preventive actions [58], antiradical and antibacterial activities [59]. In addition, it reduced the neurotoxicity in cultured dopaminergic cells induced by rotenone, and it may a possible compound to treat Parkinson’s disease [60]. 3’,5’-Cyclic Inosine monophosphate (7%), is a quinoxalinedione derivative drug which acts as a competitive antagonist of the AMPA receptor. It was investigated as an anticonvulsant for the treatment of epilepsy and as a potential treatment for neuropathic pain and cerebral ischemia [61].

Vitamin B12 or cobalamin (7.1%), is a water-soluble vitamin act as a cofactor in DNA synthesis, in both fatty acid and amino acid metabolism [62]. It is important in the normal functioning of the nervous system via its role in the synthesis of myelin [63], and in the maturation of red blood cells in the bone marrow [64]. Vitamin B12 deficiency can potentially cause severe and irreversible damage, especially to the brain and nervous system [65], symptoms such as feeling tired and weak, feeling like one may faint, staggering balance problems [66], mania and psychosis [67], pernicious anemia [68], may be weakened immunity, reduced fertility and interruption of women blood circulation [69].

Ononin (7.4%) is an isoflavone glycoside that has anti-inflammatory effects on lipopolysaccharide (LPS)-induced inflammation [70]. Aloin or barbaloin (9.6%), is a anthraquinone glycosyl from phenolic compounds [71]. used as a stimulant-laxative, treating constipation by inducing bowel movements [72].

Ferrioxamine (9.6%), is a siderophore compound indicated and determined the ferric iron in plants. Utilization of ferrioxamine E (FE) as a sole source of iron distinguishes *Salmonella* sp. from a number of related species [73]. siderophores as (68) Ga-ferrioxamine E (FOXE) used for *Aspergillus* sp. infection imaging, it have high uptake in *Aspergillus fumigates* and the higher sensitivity in vitro and in vivo [74].

B 1 (Binder) (10.1%) from the organic binders (animal and plant glues). Materials include resins, wax, linseed oil, natural gums, methyl cellulose, or proteins, are various edible thickening agents are used in cooking as binders. Some of them, e.g. various starches, tapioca flour, lactose, sucrose and microcrystalline cellulose are also used in pharmacology in making tablets as a tablet binders [54].

Rifabutin or mycobutin (10.5%), is an antibiotic in rifamycin family from amides group. It used to treat tuberculosis and prevent and treat *Mycobacterium avium* bacteria complex disease as Crohn’s disease. It is used for people with infection of human immunodeficiency virus and acquired immunodeficiency syndrome (HIV/AIDS). Also, mycobutin used with other antimycobacterial medications for active tuberculosis. In addition, mycobutin is beneficial in treating of *Chlamydophila pneumoniae* (Cpn) infection and acute liver disease [75, 76].

Vitamin K1 (13%) is phylloquinone made by plants, and found in high amounts in green leafy vegetables, because it is directly involved in photosynthesis. It is active as a vitamin in animals and performs the classic functions of vitamin K, including its activity in blood-clotting proteins production. Also, it used as a dietary supplement and to treat certain bleeding disorders as warfarin overdose, vitamin K deficiency and obstructive jaundice [33].

### Antimicrobial activities

**Fig. 3:**
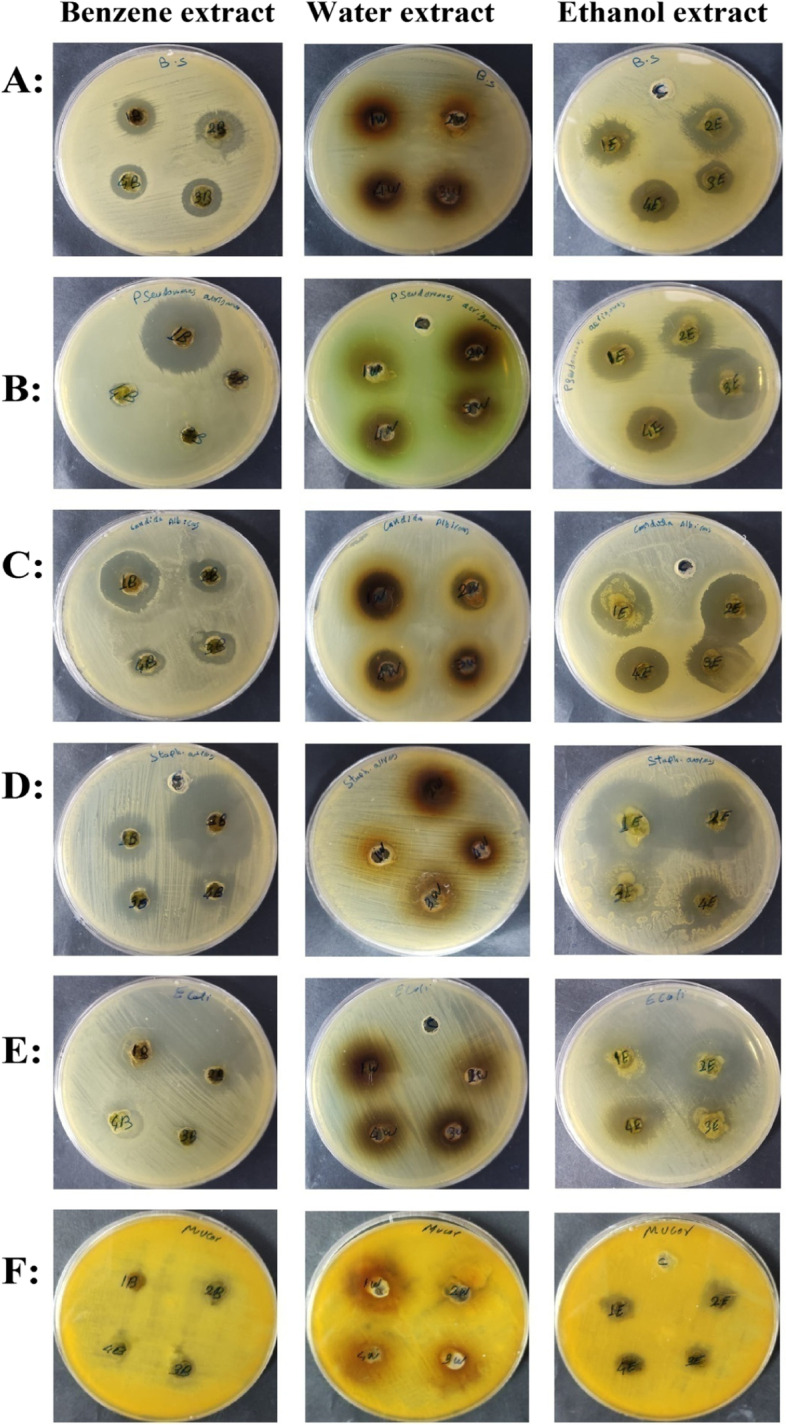
Inhibition zones of antimicrobial activities of different extracts of *Salvia* spp. against tested microorganisms. B: Benzene extract, W: Water extract, E: Ethanol extract, C: Control, 1,2 *S. aegyptiaca*, 3,4 *S. lanigera*. **A** *Bacillus subtilis*, **B** *Pseudomonas aeruginosa*, **C** *Candida albicans*, **D** *Staphylococcus aureus*, **E** *Escherichia coli*, **F** *Mucor reinelloids*

**Fig. 4:**
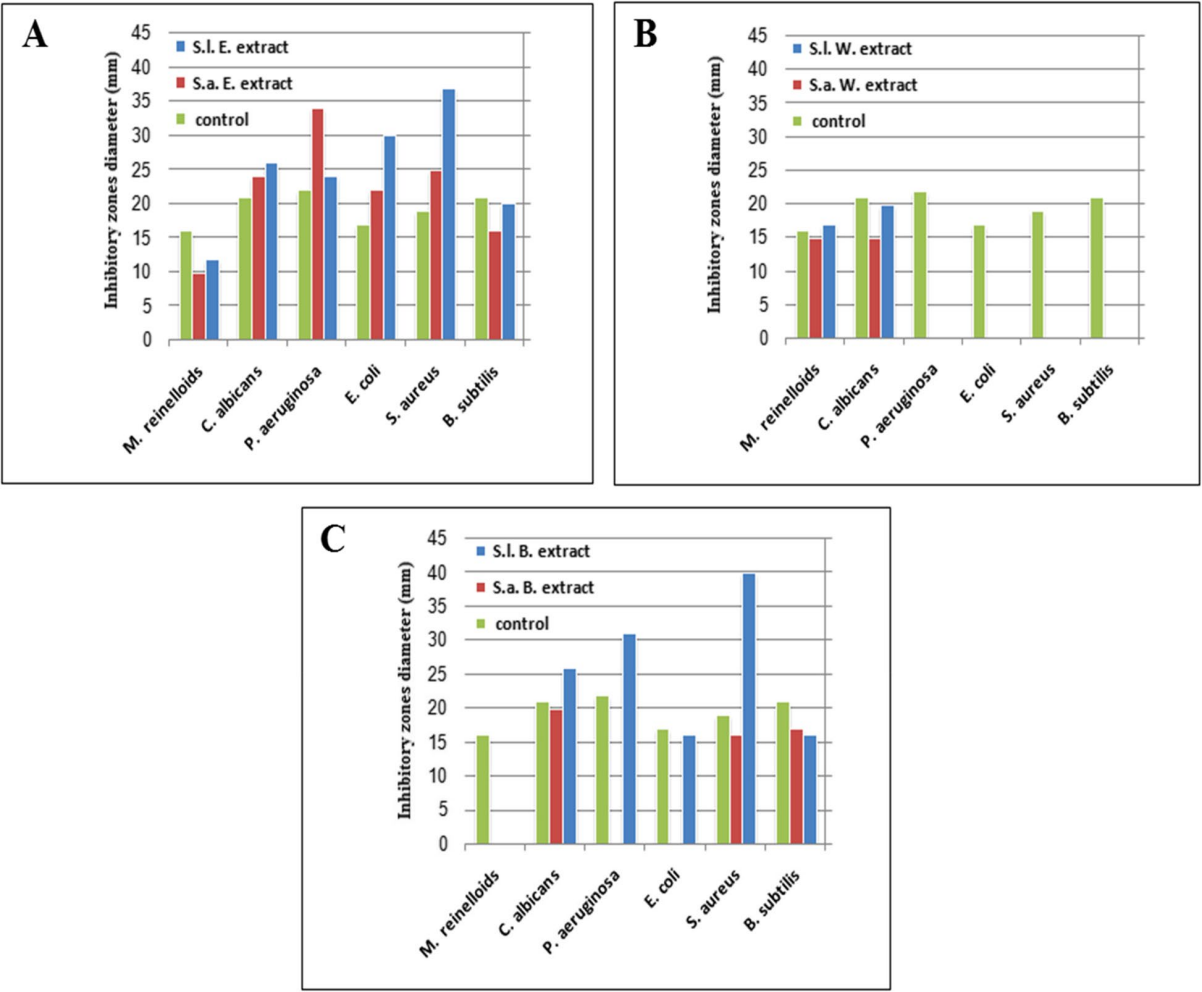
Antimicrobial effect of *S. aegyptiaca* and *S. lanigera* extracts on inhibition zone diameter of tested micro- organisms. **A** ethanol extract, **B** water extract, **C** Benzene extract, standard antimicrobial drug (Gentamycin) is the control

The antimicrobial activities of the ethanol, water and benzene extracts of the two studied species were tested against different pathogenic bacterial strains (*B. subtilis* (ATCC 6633), *P. aeruginosa* (ATCC 90,274), *E. coli* (ATCC 8739) and *S. aureus* (ATCC 6538)) and fungal strains(*C. albicans* (ATCC 10,221) and *M. reinelloids*) and compared with the standard antimicrobial drug (Gentamycin)which recorded as the most used antibiotic to treat several types of microbial infections [77]; the results are shown in (Table 3; Figs. 3 and 4).

**Table 3:** The antimicrobial activities of different extracts of studied *Salvia* spp. against pathogenic strains. Data are expressed as diameter of inhibition zone (mm). E. extract: ethanol extract, W. extract: water extract, B. extract: benzene extract

Pathogenic microorganism		*Bacillus subtilis*	*Staphylococcus aureus*	*Escherichia coli*	*Pseudomonas aeruginosa*	*Candida albicans*	*Mucor reinelloids*
		Inhibitory zones diameter (mm)					
*S. lanigera*	E. extract	20	37	30	24	26	12
	W. extract	**--**	**--**	**--**	**--**	20	17
	B. extract	16	40	16	31	26	**--**
*S.aegyptiaca*	E. extract	16	25	22	34	24	10
	W. extract	**--**	**--**	**--**	**--**	15	15
	B. extract	17	16	**--**	**--**	20	**--**
Antibacterial control (Gentamycin)		21	19	17	22	21	16

In general, ethanol extracts of the two species had the most inhibitory effect against all tested microorganisms, compared with the other two solvents (benzene, water) that had no effect upon all tested organisms. Ethanol extract not only affected all tested microorganisms but also showed bigger inhibition zones than the antimicrobial standard control (Gentamycin). Comparing the effect of ethanol extract of the two studied species, *S. lanigera* extracts showed bigger inhibition zones than *S. aegyptiaca* in all tested microorganisms except for *P. aeruginosa*. The water extracts of the two studied *Salvia* sp. had no effect against the five bacterial strains while only had effect against the two fungal strains. And, benzene extracts of the two *Salvia* species showed different antimicrobial potentials against the tested organisms.

## Discussion

LC-MS analysis detected a number of compounds within the two studied *Salvia* species that have antimicrobial properties. Two compounds with antimicrobial activity were recorded in *S. aegyptiaca*: Carnosol (5.8%) [59] and Ferrioxamine (9.6%) [74]. In *S. lanigera*, three compounds were detected: Rifabutin (10.5%) [75], 2á, 4a- Epoxymethyl phenanthrene- 7- methanol, 1,1- dimethyl- 2- methoxy- 8-(1,3- dithiin- 2- ylidene) methyl- 1,2,3,4,4a,4b,5,6,7,8,8a,9- dodecahydro-, acetate (2.4%) [78] and 2,4,6-Tris (1- (4-methoxy phenyl)- 2- morpholino) vinyl)- 1,3,5- triazine (1.1%) [79]. Moreover, LC-MS analysis revealed the presence of some metabolites groups that has a strong antimicrobial activity such as phenolics and flavonoids [80, 81]; alkaloids [82, 83] and terpenoids [84].

**Fig. 5:**
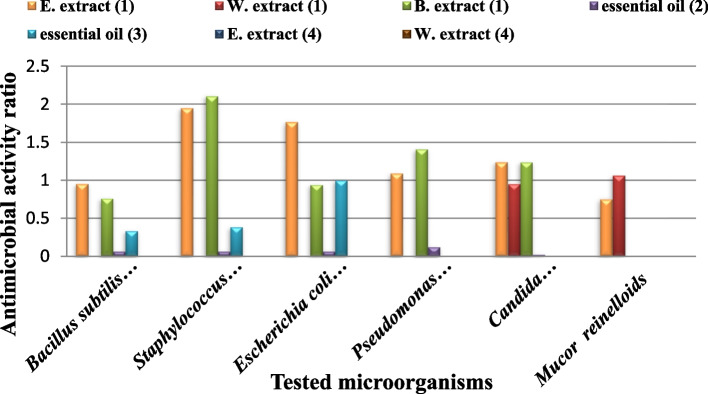
Antimicrobial activities of different *S. lanigera* extracts against tested microorganisms. E. extract: ethanol extract, W. extract: water extract, B. extract: benzene extract, **1** The present work, **2** Cyprian extracts [11], **3** Egyptian extracts [24] and **(4)** Libyan extracts [20]. The antimicrobial activity is represented by the ratio of the extract activity to the antimicrobial standard activity

Comparing the antimicrobial activity of *S. lanigera* with previous studies in (Table 4; Fig. 5), we found that the essential oil of Egyptian *S. lanigera* resulted in wider inhibition zone against *S. aureus* than that caused by the essential oil of Saudi Arabian *S. lanigera* by [22]. Moreover, Egyptian *S. lanigera* showed high antimicrobial activity against *E-coli* that was resistant to the essential oil of Saudi Arabian *S. lanigera*. On the other hand, the ethanol extract of Libyan *S. lanigera* showed antimicrobial activity lower than the antimicrobial standard against *S. aureus, E-coli* and *P. aeruginosa* [20]. While the antimicrobial activity of ethanol extract in our study was higher than the antimicrobial standard. Hence, the Egyptian *S. lanigera* showed the best antimicrobial properties against the tested microorganisms. Considering the extract type, ethanol and benzene extract in this study achieved inhibition zone wider than that of the essential oil of Egyptian *S. lanigera* tested by [24]. Moreover, the two extracts showed higher antimicrobial activities than both the Saudi Arabian and Cyprian essential oils [11, 22]. Therefore, the antimicrobial activity of *S. lanigera* may depend on two factors; the locality (habitat) and the extract type. We can say that the ethanol extract of the Egyptian *S. lanigera* exhibited the best antimicrobial properties against tested microorganism.

**Table 4:** The antimicrobial activity of *S. lanigera* different extracts in comparison with previous studies

The study	The present work				[22] Saudi Arabia		[11] Cyprus		[24] Egypt		[20] Libya		
Extract type	**E. extract**	**W. extract**	**B. extract**	**Gentamycin**	**essential oil**		**essential oil**	**Gentamycin**	**essential oil**	**Ampicillin**	**E. extract**	**W. extract**	**Streptomycin**
Method	**IZ (mm)**	**IZ (mm)**	**IZ (mm)**	**IZ (mm)**	**IZ (mm)**	**MIC (µg∕ml)**	**MIC (µg∕ml)**	**MIC (µg∕ml)**	**IZ (mm)**	**IZ (mm)**	**MIC (µg∕ml)**	**MIC (µg∕ml)**	**MIC (µg∕ml)**
*Bacillus subtilis*	20	NA	16	21	8	600	25	1.56	8	24	NT	NT	NT
*Staphylococcus* aureus	37	NA	40	19	9	450	50	3.12	10	26	20	30	0.016
*Escherichia coli*	30	NA	16	17	NA	NT	50	3.12	16	16	30	50	0.012
*Pseudomonas aeruginosa*	24	NA	31	22	NA	NT	> 100	12.5	NT	NT	35	> 50	0.016
*Candida albicans*	26	20	26	21	12	450	50	NT	NT	NT	NT	NT	NT
*Mucor reinelloids*	12	17	NA	16	NT	NT	NT	NT	NT	NT	NT	NT	NT

*E Extract *Ethanol extract, *W. extract *Water extract, *B. extract *Benzene extract, *IZ *Inhibition zone, *MIC *Minimal inhibitory concentration, *NA *Not active, *NT *Not tested

**Fig. 6:**
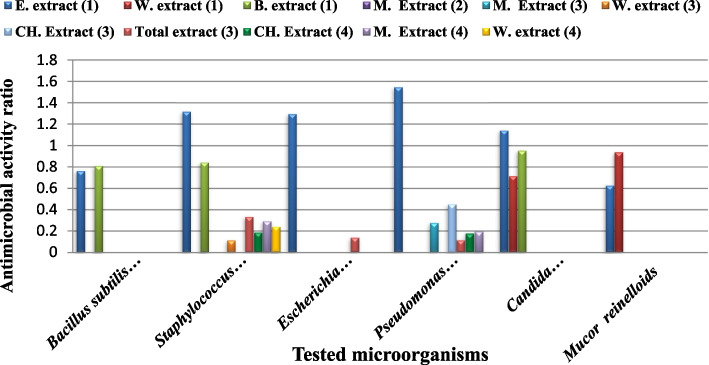
Antimicrobial activities of different *S. aegyptiaca* extracts against tested microorganisms. E. extract: ethanol extract, W. extract: water extract, B. extract: benzene extract, M. extract: methanol extract, CH. extract: Chloroform extract, **1** The present work, **2** Iranian extracts [87], **3** Iranian extracts [88] and **4** Indian extracts [89]. The antimicrobial activity is represented by the ratio of the extract activity to the antimicrobial standard activity

The antimicrobial activity of *S. aegyptiaca* extracts in the present study showed the highest antimicrobial activities than in other studies (Table 5; Fig. 6). This clear in comparing with the antimicrobial standards especially ethanol extract that had the highest antimicrobial ration against all tested microorganisms except for *B. subtilis* which was more sensitive to the benzene extract and *M. reinelloids* which was more sensitive to the water extract. These results may be attributed to the high contents of both phenolics and flavonoids in ethanolic extracts of studied *Salvia* species. However, ethanol is a perfect solvent to extract the organic compounds especially for the phenolics and flavonoids [85]. The variation in antimicrobial effects among different localities may be attributed to the variation in chemical composition that is affected by the environmental factors, as GC-MS analysis of *S. lanigera* showed a variation in detected substances: monoterpenes and thymol [86]; monoterpene hydrocarbons, oxygen containing monoterpenes, sesquiterpene hydrocarbons, acids, oxygen containing sesquiterpenes, phenolic compounds, carbonylic compounds, terpenoids, and esters hydrocarbons [11]; oxygenated sesquiterpene, cis, trans farnesol, spathulenol, caryophyllene oxide, sabinene, camphor, oxygenated monoterpene, monoterpene hydrocarbons, α-pinene, α-thujone, linalool, sesquiterpene hydrocarbons, γ-muurolene, carotol, globulol, n-alkanes, aliphatic alcohols and fatty acids [24].

**Table 5:** The antimicrobial activity of *S. aegyptiaca* different extracts in comparison with previous studies

The study	The present work				[23] Tunisia	[87] Iran		[90] Iran	[88] Iran					[89]			
Extract type	**E. extract**	**W. extract**	**B. extract**	**G**	**M. extract**	**M. extract**	**C**	**essential oil**	**M. extract**	**W. extract**	**CH. extract**	**Total extract**	**G**	**CH. extract**	**M. extract**	**W. extract**	**Streptomycin**
Method	**IZ (mm)**	**IZ (mm)**	**IZ (mm)**	**IZ (mm)**	**MIC (µg∕ml)**	**MIC (µg∕ml)**	**MIC (µg∕ml)**	**MIC (µg∕ml)**	**Cup Plate**	**Cup Plate**	**Cup Plate**	**Cup Plate**	**Cup Plate**	**IZ (mm)**	**IZ (mm)**	**IZ (mm)**	**IZ (mm)**
*Bacillus subtilis*	16	NA	17	21	NT	NA	0.0125	NT	NT	NT	NT	NT	NT	NT	NT	NT	NT
*Staphylococcus aureus*	25	NA	16	19	NT	NA	0.0125	128	NA	4	NA	11.6	35	0.93	1.45	1.2	5
*Escherichia coli*	22	NA	NA	17	NT	NA	0.05	NT	NA	NA	NA	5.3	38	NT	NT	NT	NT
*Pseudomonas aeruginosa*	34	NA	NA	22	> 1000	NT	NT	NT	11	NA	18	4.6	40	1.23	1.36	NA	7
*Candida albicans*	24	15	20	21	> 1000	NT	NT	NT	NA	NA	NA	NA	NT	NT	NT	NT	NT
*Mucor reinelloids*	10	15	NA	16	NT	NT	NT	NT	NT	NT	NT	NT	NT	NT	NT	NT	NT

*E. extract *Ethanol extract, *W. extract *Water extract, *B. extract *Benzene extract, *M. extract *Methanol extract, *CH. extract *Chloroform extract, *IZ *Inhibition zone, *MIC * minimal inhibitory concentration, *G *Gentamycin, *Cp *Chloramphenicol, *NA *Not active, *NT *Not tested

At the same time, *S. aegyptiaca* was reported to contain an ursolic acid, oleanolic acid, α-amyrin, β -amyrin, 3β- hydroxy- oleana- 11,13 (18- dien- 28- oic acid, 12-ursadien-22-oic acid and lupeol [91]; tricyclene, limonene, β-pinene, caryophyllene oxide and β-caryophyllene [92]. In addition, aegyptinones A and B, 6-methylcryptoacetalide, 6-methylcrypto- tanshinone and 6-methylepicryptoacetalide [93].

## Conclusions

The total phenolics in *S. lanigera* and *S. aegyptiaca* were 132.61 ± 6.23 and 125.19 ± 4.97 mg/g, respectively. While the total flavonoids were 35.68 ± 1.84 and 40.63 ± 2.11 mg/g, respectively. Through LC-MS analysis, twenty six metabolites were identified in *S. aegyptiaca* extract; three terpenoids, one flavonoid, seven phenolics, eight alkaloids, four steroids, one mercaptans, one amine compound and one fatty acid; while eighteen metabolites were identified in the extract of *S. lanigera;* one terpenoids, two flavonoids, six phenolics, five alkaloids, one mercaptans and three amine compounds. Two compounds were detected in both species; heptadecanoyl coenzyme A; a dervitative of the fatty acid heptadecanoic acid that represent a large peak area of 13.5% in *S. aegyptiaca* and 11.5% in *S. lanigera*. Oenin; a pigment that represent a peak area of 3.1% in *S. aegyptiaca* and 1.2% in *S. lanigera*. Ethanol extracts of both *Salvia* spp. had the most inhibitory effects against all tested microorganisms that exceeded the effect of the standard, except for *Mucor reinelloids* which was more sensitive to the water extract. Moreover, *S. lanigera* ethanol extract showed larger inhibition zone than *S. aegyptiaca* with all tested micro- organisms except for *Pseudomonas aeruginosa*. However, the water extracts of both *Salvia* spp. had no effect against the tested bacterial strains and only inhibited the growth of the tested fungal strains. The antimicrobial activity of *Salvia* spp. may be attributed to two factors; the locality and the type of extraction solvent. Moreover, we can say that, the ethanol extract of the Egyptian *S. lanigera* had the best antimicrobial properties against the tested microorganisms as compared with many previous studies. The antimicrobial potentials may be credited to the high phenolics & flavonoids contents in *Salvia* spp. ethanol extracts.

## Materials and methods

### Species collection and identification and sampling for analysis

Fresh plant materials of the two studied species *S. aegyptiaca* and *S. lanigera* were collected from the same natural habitat (roadsides in Marsa-Matrouh, Egypt). The collected materials (Apr. 2021) were matched to the herbarium sheets of *Salvia* species deposited at Botany Department Herbarium, Faculty of Science, University of Cairo(CAI) And Botany Department Herbarium, Faculty of Science, University of Menoufia in Egypt, given a voucher numbers; SL1122 AND SE3344 for *S. lanigera* and *S. aegyptiaca*, respectively. The identification of the two species was confirmed by expert taxonomists; Prof. Zaki Turki and Dr. Ann Abozeid.

A number of fresh samples in the maturity stage were collected from the same habitat within the study area (to compare the two studied species). Samples were air-dried then milled to fine uniform texture homogenized by grinding in stainless steel blender and kept in small paper bags or stored in tubes.

### Plant sampling for LC-mass analysis

5gm powdered plants were homogenized then macerated in a stoppered container with 100 ml 85% methanol and allowed to stand at room temperature for a period of 24 h. for conventional extraction, the extract and powdered were placed in a sonicator at 40 ˚C for 60 min. Then this extract was filtered and concentrated under vacuum at 40 ˚C by using Rota vapor to provide crude extract (gm).

### Extraction procedures

The solvents used for extraction of biological study were benzene, distilled water and ethanol (70%). 25 g of dried and powdered plant material were packed in each one of the three solvents and extracted with separately different solvents. For each extract, the solvent was removed by evaporation under low pressure using an evaporator at a temperature not exceeding 50 ± 2 °C, and then one fraction for each solvent was collected [94]. The crude extract is dissolved in 1 ml of each one of the three solvents to applied on the used microorganisms.

### Phytochemical investigations

#### Total phenolic content

Total phenolic content (TPC) was determined using the Folin-Ciocalteu colorimetric method [95]. A volume of 3mL of Folin-Ciocalteau (10%) mixed with five µL (0.05mL) plant extract and 0.8mL sodium bicarbonate (7.5%). The reaction solution was incubated at room temperature for 30 min. The absorbance of the mixture was measured at 765 nm. By means of Milton Roy (Spectronic 1201) spectrophotometer. The TPC was expressed as mg gallic acid equivalents (GAE)/g extract.

#### Total flavonoids content

Total flavonoids Content (TFC) was quantified by using this method: briefly, 0.1mL extract was mixed with 3.90mL distilled water and with 0.3mL sodium nitrite (5%) solution, allowed to react for 5 min. Then 0.3mL aluminum chloride (10%) solutions was added. The mixture was allowed reacting further for 6 min. After this time, the mixture solution was treated with 2mL of 1mM^− 1^ sodium hydroxide. Finally, 2.4mL distilled water was added to all samples. The absorbance was read at 510 nm against a sample blank without reaction using Milton Roy (Spectronic 1201) spectrophotometer. The TFC of the extracts were expressed as mg quercetin equivalents (QE) /g extract [96].

#### LC-MS analysis

The separation and identification of the pure active materials from plant parts carried by using LC-MS system (UHPLC-TSQ Quantum Mass Spectrometer). The chemical composition of the pure active materials from plant samples were performed using Regular Method, ionization mode (–ve), electron scattering impact (ESI), MS Run Time (46.00 Segment)(min), Scan Events (1: - c Full Q1MS, Micro Scans 1), Scan Time (1.00, Q1), PW 0.70, [100.000-1000.000] and program for Dionex (UHPLC), in labs of fungi center in Al-Azhar university. The nominal temperature was 25 °C, lower limit temperature 23 °C and upper limit temperature 27 °C. The equilibration time was 0.5 (min). Ready temperature delta was 1.0 °C and upper limit pressure was 1034 (bar). Maximum flow ramp down was 6.000 (ml/min²), when maximum flow ramp up was 6.000 (ml/min²). Sample height was 2.000 (mm). And inject wash was after draw with wash volume 100.000 (µl), wash speed 10.000 (µl/s) and loop wash factor 2.000. From the property to start the run or injection was the wait start run flow 0.500 (ml/min). The components were identified by comparison of their retention times and mass spectra with those of WILEY 09 and NIST 11 mass spectral database, all detected compounds with their chemical structure and fragments are listed in Supplementary Table 1.

### Biological study

#### Microorganisms used

The following microorganisms obtained from culture unit in the Regional Centre for Mycology and Biotechnology (RCMB), Al-Azhar University: *Bacillus subtilis* (ATCC6633), *Escherichia coli* (ATCC8739), *Staphylococcus aureus* (ATCC6538) & *Pseudomonas aeruginosa* (ATCC90274) and fungal strains: *Candida albicans* (ATCC10221) and *Mucor reinelloids*. The bacterial and fungal strains were cultured in nutrient agar and malt extract, respectively.

#### Method of agar disk diffusion

Antimicrobial activity was determined by using agar disk diffusion method, by using of filter paper discs in 6.0 mm (100 ul was tested) diameter. About 10 mg/ml of all samples were dissolved in normal saline (0.9% NaCl) or DMSO. Gentamycin is the substance that used as a control. Agar disk diffusion method employs an agar plates that should be inoculated within 15 min after adjusting the suspension. The entire dried agar surface was evenly streaked in three different directions.

Let the agar surface to dry for 15 min, Firstly, inoculated the agar plate surface by spreading a volume of the microbial inoculums over the entire agar surface. Then, dipping the discs in (20–100mL) of the antimicrobial agent or extract solution at desired concentration. After that, placing the discs with a diameter of 6.0 mm (100 ul was tested) aseptically with a sterile forceps in agar plates, that were incubated under suitable conditions depending upon the test microorganism (about 15 min). The antimicrobial agent diffuses in the agar medium to inhibit the growth of the tested microbial strain [97].

After incubation times of 16 to 24 h (Mucoraceae), 24 h (*A. fumigatus*, *A. flavus*, *A. niger*) or 48 h (other species), the resulting inhibition zone diameters (in mm) surrounding the wells should be measured to the nearest whole millimeter at the point at which there is prominent reduction in growth. If growth is insufficient at the recommended times, the plates should be re-incubated and read later [98].

### Statistical analysis

One-way analysis of variance (ANOVA 1) was used, after testing the data for normality, to assess the variation in soil, plant variables and phytochemical constituents among the different habitats of studed *Salvia* sp. according to SPSS software (2006). A post-hoc test was applied according to (Duncan’s test) when differences are significant.

### Used chemicals

The companies names, cities and countries of used chemicals as followed: methanol (85%), benzene, distilled water and ethanol (70%) from CNW Technologies GmbH (Dusseldorf - Germany). Aluminum chloride from Sangon Biotech (Shanghai- China). Sodium nitrite, sodium bicarbonate and sodium hydroxide from Research Lab Fine Chem. Industries (Maharashtra - India). Dimethyl sulfoxide (DMSO) from Piochem (Giza - Egypt). Gentamycin, when agar from Himedia (Giza - Egypt). Folin-Ciocalteau from Sigma Aldrich (USA). The speacies was authenticated at fungi center, Al-Azhar University (Nasr city - Egypt).

## Supplementary Information

**Additional file 1.**

## Data Availability

All data are included in the manuscript, any additional information needed contact the corresponding author.

## Abbreviations

*B. extract*: benzene extract
*C*: Control
*CH. extract*: Chloroform extract
*Cp*: Chloramphenicol
*E. extract*: ethanol extract
*G*: Gentamycin
*IZ*: inhibition zone
*M. extract*: methanol extract
*MIC*: minimal inhibitory concentration
*M.wt.*: molecular weight
*NA*: Not active
*NT*: Not tested
*S.a.*: *Salvia aegyptiaca*
*S.l.*: *Salvia lanigera*
*RT*: Retention time
*W. extract*: water extract.
