# Supplementary material for: LC-MS metabolomics profiling of Salvia aegyptiaca L. and S. lanigera Poir. with the antimicrobial properties of their extracts
Source: BMC Plant Biol. 2023 Jun 26;23:340. doi: 10.1186/s12870-023-04341-5 (PMC10291801; doi:10.1186/s12870-023-04341-5)
Supplement: Supplementary file 1 — Additional file 1. [file 12870_2023_4341_MOESM1_ESM.docx]

| **Supplementary table1: detected compounds with their fragmentations** | | | | | | | | |
| --- | --- | --- | --- | --- | --- | --- | --- | --- |
| **Compound name** | **Peak area%** | | **RT** | | **M. formula** | **M.wt.** | **Chemical structure** | **Fragmentation** |
|  | ***S.a.*** | ***S.l.*** |  |  |  |  |  |  |
| **Terpenoids** | | | | | | | | |
| Phenazine,2,2'-(3,7,12,16-tetramethyl-1,3,5,7,9,11,13,15,17-octadecanonaene-1,18-diyl)bis[3,4-dihydro-1,3,3-trimethyl-, (all-E)- | - | **6** | 13.38 | | C52H56N4 | 736 | 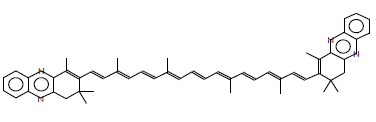 | 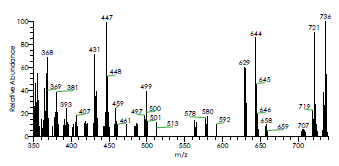 |
| Carnosol | **5.8** | - | 20.64 | | C20H26O4 | 330 | 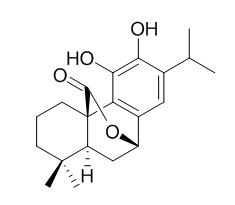 | 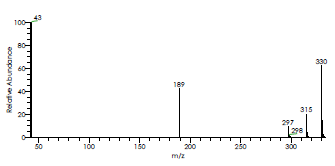 |
| Psi.,.psi.-Carotene,3,3',4,4'-tetradehydro-1,1',2,2'-tetrahydro-1,1'-dimethoxy-2,2'-dioxo- | 4.9 | - | 20.75 | | C42H56O4 | 624 | 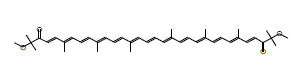 | 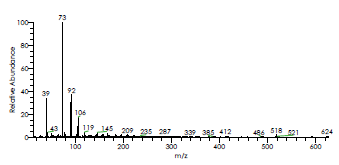 |
| **Flavonoieds** | | | | | | | | |
| Ononin | **7.4** | - | 16.3 | | C22H22O9 | 430 | 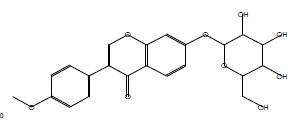 | 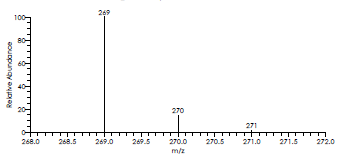 |
| Ginkgetin | - | 3.5 | 13.89 | | C32H22O10 | 566 | *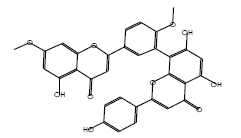* | *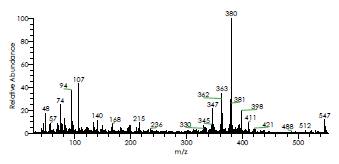* |
| **Phenolics** | | | | | | | | |
| Vitamin K1 | **13** | - | 1.47 | | C31H46O2 | 450 | 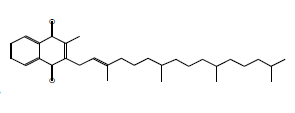 | 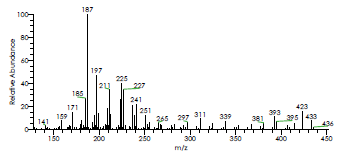 |
| Aloin | **9.6** | - | 29.32 | | C21H22O9 | 418 | 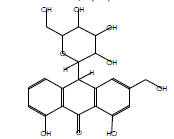 | 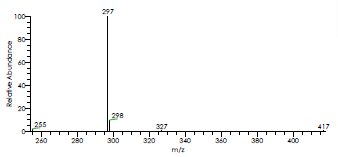 |
| Pentacontanoic acid, propyl ester | - | 3 | 21.81 | | C53H106O2 | 774 | 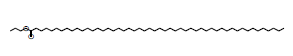 | 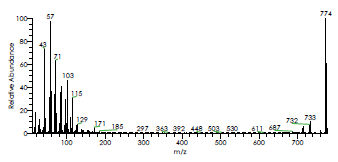 |
|  |  |  | 34.63 | |  |  |  |  |
|  |  |  | 34.98 | |  |  |  |  |
|  |  |  | 3.42 | |  |  |  |  |
| **Malvidin 3-O-galactoside cation or Oenin** | 3.1 | 1.2 | 22.26 | | C23H25O12 | 493 | **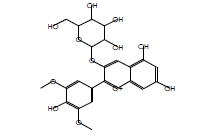** | **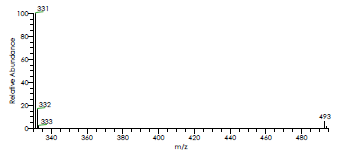** |
|  |  |  | 22.32 | |  |  |  |  |
| 2á,4a-Epoxymethyl phenanthrene-7-methanol,1,1-dimethyl-2-methoxy-8-(1,3-dithiin-2-ylidene) methyl-1,2,3,4,4a,4b,5,6,7,8,8a,9-dodecahydro-, acetate | - | 2.4 | 17.67 | | C27H38O4S2 | 490 | 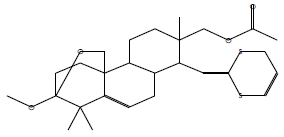 | 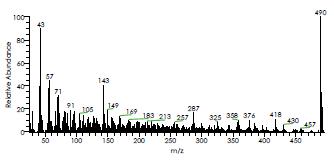 |
| Pentacontanoic acid, ethyl ester | 1 | - | 22.38 | | C52H104O2 | 760 | 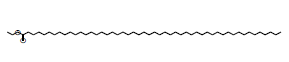 | 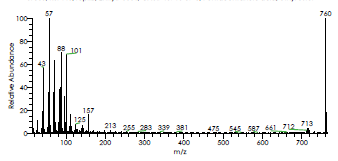 |
| Leucrose, Perboryliert | - | 0.8 | 37.61 | | C44H94B8O11 | 886 | 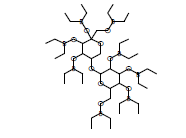 | 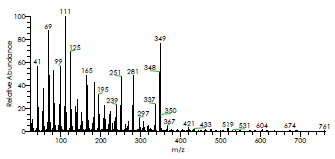 |
| Olein, 3-palmito-2-stearo-1- | - | 0.5 | 24.42 | | C55H104O6 | 860 | 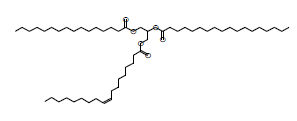 | 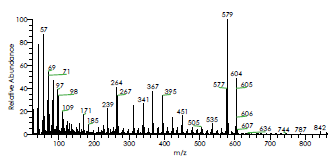 |
| **Alkaloids** | | | | | | | | |
| 1,2-Di(2,3,7,8,12,13,17,18-Octaethyl-7,8-dihydro-21H,23H-porphinyl-10)ethane | - | **10.19** | | 19.9 | C74H98N8 | 1098 | 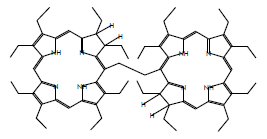 | 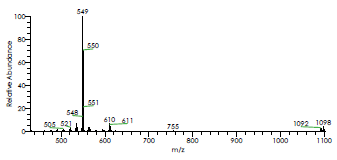 |
| 6à,7à:14,7á-Bis(oxymethylene)-4,5à-epoxy-17-methylmorphinan-3-ol | - | **5.4** | | 15.56 | C19H21NO4 | 327 |  | 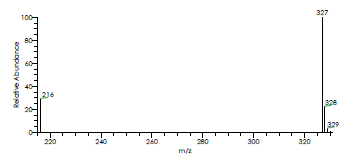 |
| Acepromazine | 4.1 | - | | 15.62 | C19H22N2OS | 326 | 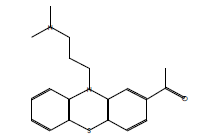 | 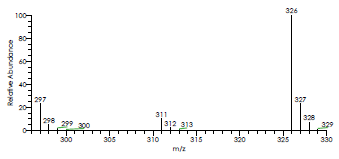 |
| 5-Isoxazolidinol, 2,3-diphenyl-,acetate (ester), cis | - | 3.6 | | 18.45 | C17H17NO3 | 283 |  | 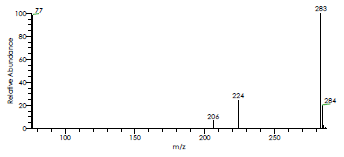 |
| 3-[7,12-Bis-(2-hydroxy-ethyl)-18-(2-methoxycarbonyl-ethyl)-3,8,13,17-tetramethyl-porphyrin-2-l]-propionic acid methyl ester | 2.6 | - | | 14.42 | C36H42N4O6 | 626 | 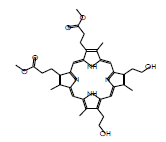 | 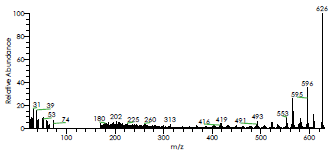 |
| Dimethyl-fenbend azole | 2.1 | - | | 15.56 | C17H17N3O2S | 327 |  | 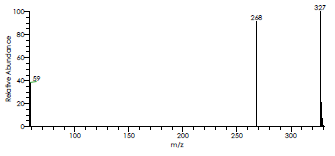 |
| 2-Benzyl-3-(4-methoxyphenyl)-3,4,4a,7-tetrahydroisoquinolin-1(2H)-one | - | 1.6 | | 14.46 | C23H23NO2 | 345 |  | 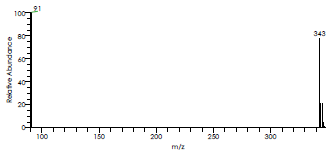 |
| Tetrandrine | 1.1 | - | | 19.94 | C38H42N2O6 | 622 | *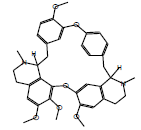* | *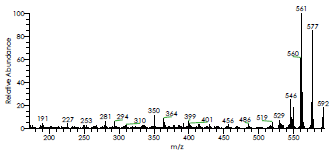* |
| Wilforine | 0.7 | - | | 0.13 | C43H49NO18 | 867 | 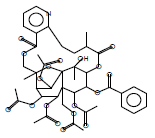 | 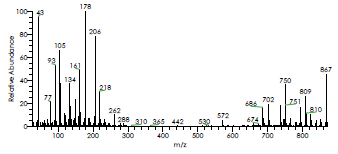 |
|  |  |  |  | 3.09 |  |  |  |  |
|  |  |  |  | 6.46 |  |  |  |  |
| **Steroids** | | | | | | | | |
| Cholest-7-en-6-one, 2,3,14,20,22,25-hexahy  droxy-, (2á,3á,5á,22R)- | 2.6 | - | 17.35 | | C27H44O7 | 480 | *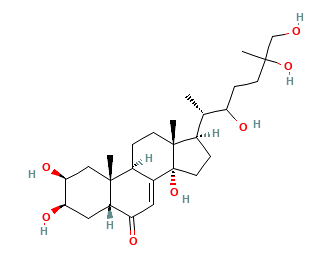* | *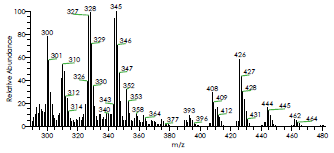* |
| 5à-Cardanolide, 3á,14,19-trihydroxy- | 0.85 | - | 38.16 | | C23H36O5 | 392 | 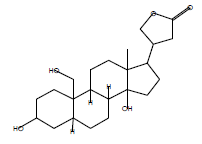 | 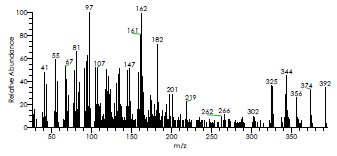 |
| **Mercaptans** | | | | | | | | |
| **Heptadecanoyl coenzyme A** | **13.5** | **11.5** | 33.9 | | C38H68N7O17P3S | 1019 | 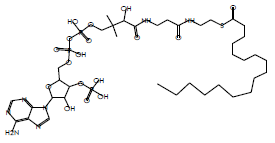 | 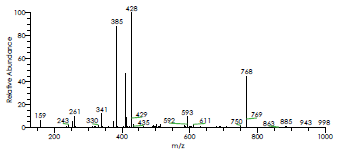 |
| **Amine compounds** | | | | | | | | |
| B 1 (BINDER) | - | **10.1** | 23.66 | | C72H124N4O17 | 1316 |  | 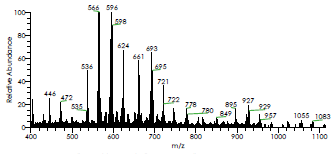 |
| Rifabutin | - | **10.5** | 23.26 | | C46H62N4O11 | 846 | 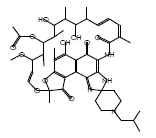 | 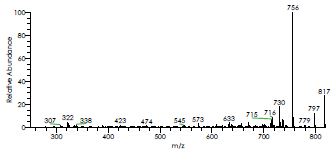 |
| Ferrioxamine | **9.6** | - | 23.85 | | C27H45FeN6O9 | 653 | 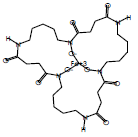 | 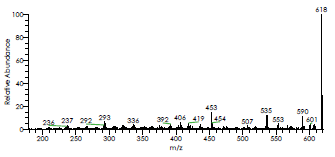 |
| 3',5'-Cyclic Inosinemonophosphate | - | **7** | 16.01 | | C10H11N4O7P | 330 | 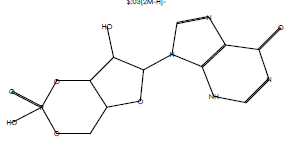 | 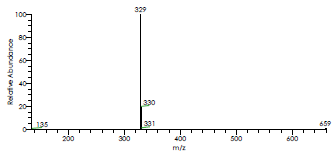 |
| Vitamin B12 | **7.1** | - | | 23.76 | C63H88CoN14O14P | 1354 | 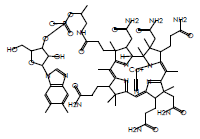 | 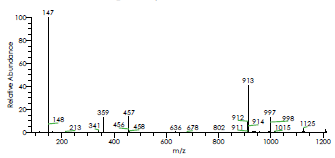 |
